# Paracrine signalling between keratinocytes and SVF cells results in a new secreted cytokine profile during wound closure

**DOI:** 10.1186/s13287-023-03488-0

**Published:** 2023-09-19

**Authors:** Stefan Balko, Evan Kerr, Ed Buchel, Sarvesh Logsetty, Afshin Raouf

**Affiliations:** 1 Department of Immunology, Rady Faculty of Health Sciences, University of Manitoba, Winnipeg, MB Canada; 2 Department of Surgery, Rady Faculty of Health Sciences, University of Manitoba, Winnipeg, MB Canada; 3 Max Rady College of Medicine, Rady Faculty of Health Sciences, University of Manitoba, Winnipeg, MB Canada; 4 CancerCare Manitoba Research Institute, CancerCare Manitoba, Winnipeg, MB Canada

**Keywords:** Wound healing, Scratch assay, Cell migration, Keratinocytes, Fibroblasts, Cytokines

## Abstract

Stromal vascular fraction (SVF) cells, and the adipose-derived mesenchymal stem cells they contain, have shown enhanced wound healing in vitro and in vivo, yet their clinical application has been limited. In this regard, understanding the mechanisms that govern SVF-enhanced wound healing would improve their application in the clinic. Here, we show that the SVF cells and keratinocytes engage in a paracrine crosstalk during wound closure, which results in a new cytokine profile that is distinct from the cytokines regularly secreted by either cell type on their own. We identify 11 cytokines, 5 of which are not regularly secreted by the SVF cells, whose expressions are significantly increased during wound closure by the keratinocytes. This new cytokine profile could be used to accelerate wound closure and initiate re-epithelialization without the need to obtain the SVF cells from the patient.

**Supplementary Information:**

The online version contains supplementary material available at 10.1186/s13287-023-03488-0.

## Introduction

Cutaneous wounds typically heal through a complex and interconnected series of steps that include homeostasis, inflammation, proliferation and finally tissue repair and regeneration. However, in the case of severe burns and other skin wounds, healing typically results in hypertrophic scar tissue formation, that leads to loss of function and deformities. In other cases, the healing of severe wounds could be stalled in one of the four healing steps, and ultimately develop into chronic non-healing wounds [1–3]. Hypertrophic scarring, and the development of non-healing wounds, significantly reduces the quality of life for patients [4]. The majority of treatment options for non-healing wounds are aimed at managing the wound bed. In some cases, skin grafts are used to encourage tissue repair and regeneration. However, the application of skin grafts to large portions of a patient’s body is not always feasible [4] and can lead to extensive scarring. To this end, mesenchymal stem cells (MSCs) have been explored for their potential to induce wound repair and tissue regeneration [5]. Typically bone marrow-derived stem cells (BMSCs), and more recently adipose-derived stem cells (ADSCs), which are enriched in the stromal vascular fraction (SVF) of fat tissue, have been used as sources of MSCs. SVF cells are a more attractive source of MSCs than bone marrow because fat grafts are more easily obtainable, and the frequency at which MSCs are found within SVF samples are higher as compared to BMSCs. Additionally, ADSCs have been shown to have better regenerative potential and immunomodulatory properties than the BMSCs, making them more suitable for stem cell therapies in the clinic [6–12].

Currently, markers that allow ADSCs to be obtained from the SVF cells at high frequencies are not available. However, in vitro and in vivo data support the beneficial effects of SVF cells on wound healing. The clinical applicability of SVF cells has unfortunately been limited because the procedures for obtaining SVF samples are time consuming, complicated and not always possible in a clinical setting. As well, the heterogeneity of the SVF samples makes it difficult to predict which patients would benefit from autologous SVF wound healing therapy. The identification of SVF-secreted factors during wound healing and wound closure [13, 14] would allow the application of the beneficial properties of SVF cells, including the ADSCs, while eliminating the requirement to obtain these cells from the patient.

In this report, we provide evidence that a paracrine crosstalk takes place between SVF cells and primary human keratinocytes during wound closure, which results in a new cytokine profile during wound healing, that is different from either keratinocyte-alone or SVF-alone cultures.

## Materials and methods

A detailed outline of the materials and methods used in this study is provided in the Additional file 1.

## Results

### The crosstalk between HEKa and P0SVF cells accelerates wound closure faster than P0SVF-CM

**Fig.1:**
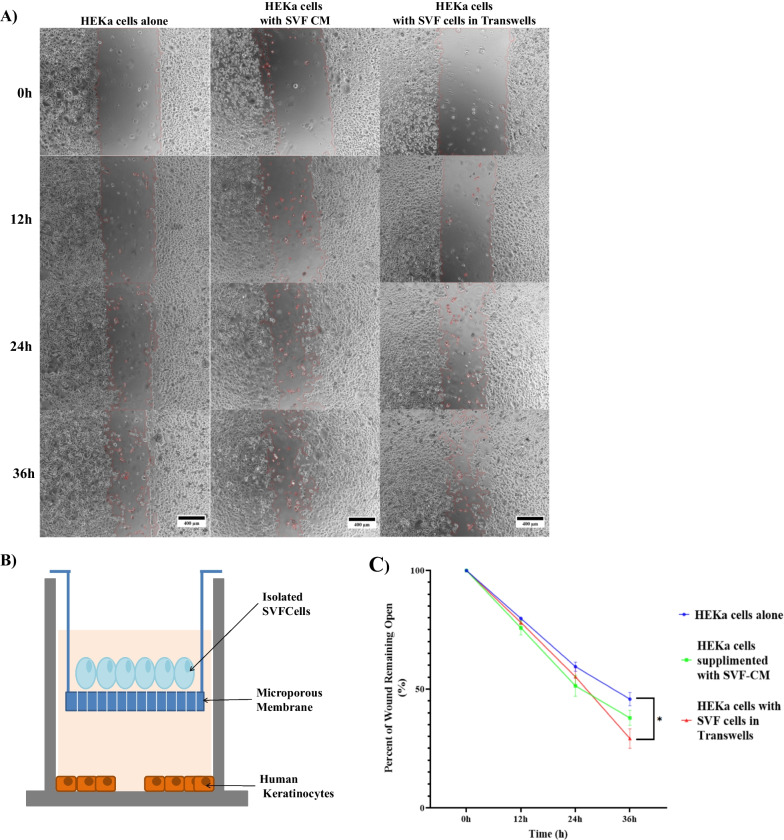
Keratinocyte wound closure was measured every 12 h, over a 36 h period, and images were taken in order to analyse wound closure. **A** representative images of scratch assays performed with HEKa-alone, P0SVF-conditioned media (CM) and the HEKa cells with SVF cells in transwell inserts, after 36 h are shown. All images taken at the same magnification and representative scale bars are show. **B** Schematic representation of the transwell insert co-culture. It should be noted that although the cells are in the same well, the microporous membrane does not allow for any cell–cell contact. **C** Wound closure over the 36 h period, represented as a line graph. Mean ± SEM, N = 3–5; *=*p*<0.05

To examine if the communication between HEKa cells and P0SVF cells has a larger impact on keratinocyte wound closure than the proteins regularly secreted by the SVF cells, we used transwell inserts in the scratch assays. These inserts allow for cell–cell communication through secreted factors, while preventing physical contact between P0SVF cells and keratinocytes (Fig. 1A,B). For this purpose, we compared the amount of wound closure by HEKa cells supplemented with either P0SVF-conditioned media (P0SVF-CM), or with P0SVF cells grown on the transwell inserts, to HEKa cells alone as controls. SVF cells were shown to be able to differentiate into the three mesenchymal lineages, showed an ADSC frequency of 1–3%, and were metabolically active (Additional file 2). We found that the keratinocytes maintained cell shape and proliferative activity in the HEKa scratch medium as compared to their respective growth medium (Fig. 1A). We also found that HEKa-alone controls had 46 ± 2.8% of the original scratch area remaining open when cultured in HEKa scratch medium (Fig. 1C). The P0SVF cells in transwell inserts significantly enhanced wound closure as compared to HEKa cells alone where only 29 ± 4.1% of the scratched area remained open after 36 h (Fig. 1C). However, the P0SVF-CM failed to significantly improve wound closure, as compared to HEKa cells alone, with 38 ± 3.2% of the scratch remaining open (Fig. 1C). No improvement in wound closure was observed at earlier time points.

### P0SVF and HEKa cells have a unique up regulation of secreted cytokines during wound closure

**Fig. 2:**
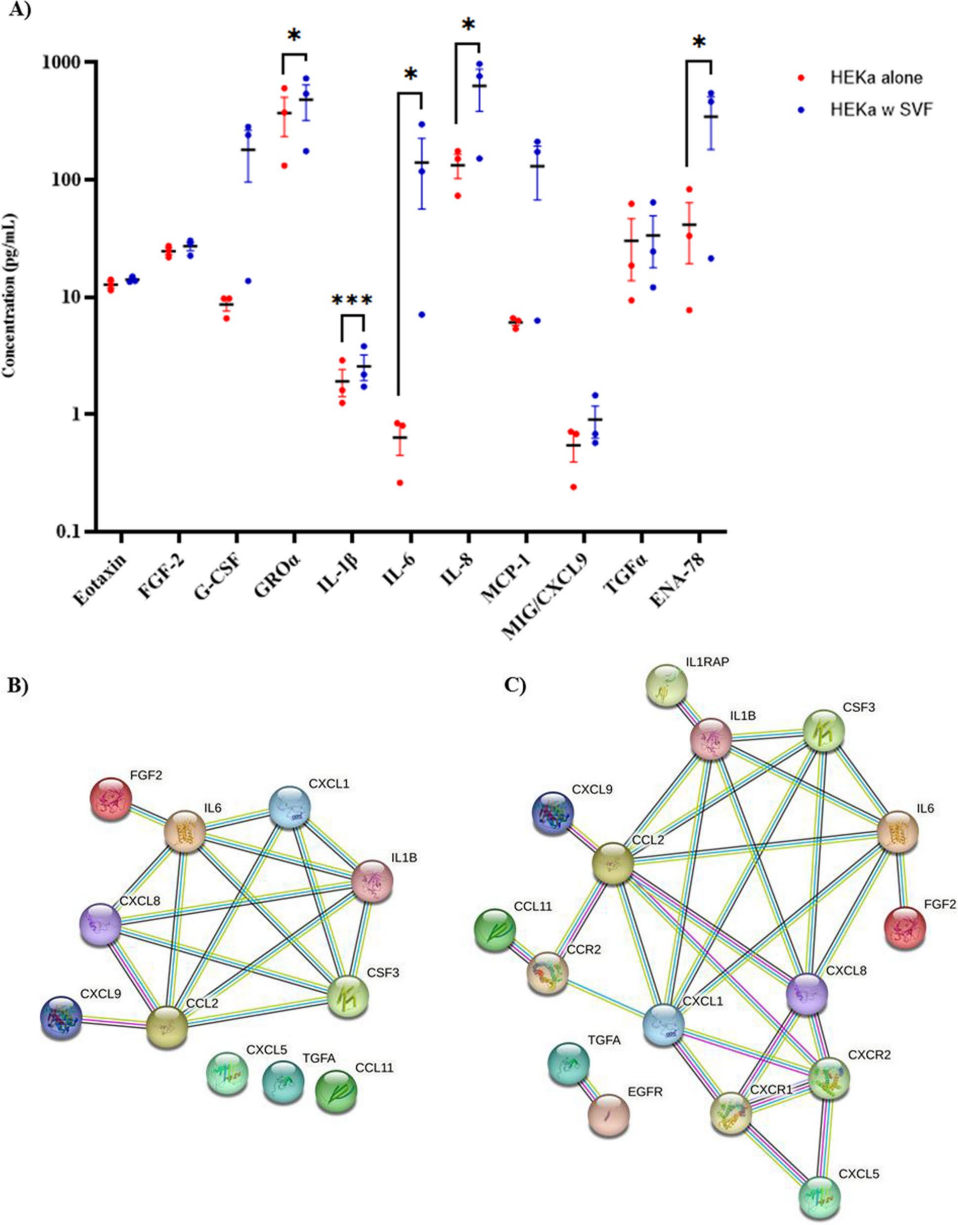
Upregulated cytokines found in the media taken from HEKa scratch experiments with SVF cells in the transwell inserts, as compared to HEKa-alone controls. **A** scatter plot showing measured concentrations in the media, blue represents HEKa cells with SVF in transwell inserts and red represents HEKa cells alone control. Of the 11 upregulated cytokines shown, 5 have shown to be statistically significant as compared to control, and the remaining 6 are statistically trending towards significance (0.1 < *p* < 0.05). Data represented as Mean ± SEM; N = 3; *=*p*< 0.05, ***=*P* < 0.001. **B** STRING protein network of the 11 cytokines found to be unregulated in the analysed media with **C** receptors for the cytokines added; connections defined as following: yellow lines = textmining, light blue lines = from curated database, violet lines = experimentally determined, black lines = co-expression

Our data indicates that the presence of the P0SVF cells in the transwell inserts is needed in order to significantly accelerate HEKa cell wound closure. In addition, factors regularly secreted by the P0SVF cells (P0SVF-CM) on their own were not sufficient to recapitulate this accelerated wound closure. We therefore hypothesized that the communication between SVF cells and HEKa cells results in a new secreted factor profile. To test this hypothesis, we collected and analysed growth media from the scratch assays. P0SVF/HEKa transwell medium, P0SVF-alone-CM and the HEKa-alone-CM were analysed for the presence of 71 different cytokines and chemokines. Cytokines found in the P0SVF/HEKa transwell medium, whose concentrations were significantly increased during wound closure, as compared to the P0SVF-CM and HEKa-CM, were identified for further analysis (Fig. 2A and Additional file 3). Such analysis revealed 11 different cytokines, 5 of which showed statistically significant elevated levels during wound healing, while the other 6 were trending towards statistical significance (Fig. 2A). Very interestingly, of the 11 cytokines found, five were not detectable in the P0SVF-CM (G-CSF, GROα, ENA-78, TGFα and CXCL9) (Additional file 3). Moreover, G-CSF, IL-6 and MCP-1 were present at very low levels in the HEKa-alone-CM (< 10 pg/mL) but were found at very high levels in P0SVF/HEKa transwell medium. Among these 11 cytokines, IL-6 concentration was increased the most by 221.9 fold, followed by MCP-1and G-CSF (21.3 fold and 20.7 fold, respectively) during P0SVF-enhanced wound closure. Using a human functional protein association network, we found that all of the upregulated cytokines, with the exception of ENA-78 (CXCL5), TGFα and Eotaxin (CCL11), formed an interconnected signalling network (Fig. 2B). Signalling through IL-8 (CXCL8), ENA-78 (CXCL5), GROα (CXCL1) and MCP-1 (CCL2) all activate the CXCR2 receptor (Fig. 2C). IL-8 and GROα share signalling through CXCR1, while MCP-1 and GROα share signalling through CCR2 (Fig. 2C).

## Discussion

Previous studies have examined the role of secreted proteins by SVF cells conditioned media in wound healing, using keratinocytes or dermal fibroblasts, and identified a few cytokines with positive effects on wound healing (e.g. IL-8) [15–19]. In this study, however, we found 5 cytokines that are not regularly secreted by the P0SVF cells, but their expression was significantly increased during P0SVF-enhanced wound closure. As well, we show that the conditioned media obtained from the P0SVF-CM is not as effective in enhancing wound closure compared to HEKa wound closure in the presence of SVF cells, where physical interactions between keratinocytes and SVF cells was inhibited, but the free exchange of secreted cytokines, growth factors and extracellular vesicles was permitted. Previous studies have demonstrated enhanced wound closure with SVF-CM [19], whereas in our hand, we observed a positive trend in HEKa cell wound closure that was not statistically significant. This discrepancy could be due to the different growth medium we used (HEKa scratch media) and that these previous studies used fibroblast or keratinocyte cell lines, whereas here, we used primary human SVF and minimally passaged primary human keratinocytes (HEKa cells). It is noteworthy that that here we used a lineage cell depleted (CD45^+^CD31^+^-removed) subset of SVF cells that are devoid of endothelial progenitor cells. It would be interesting to examine the potential contribution of these endothelial progenitors to the SVF-enhanced keratinocyte wound closure.

In efforts to study the altered profile of secreted cytokines during wound closure, one set of CM was obtained from scratches that reached > 80% wound closure in 24 h compared to the other two data sets that were obtained from scratches that closed in 36 h. Although one set of scratches closed faster, they showed similar altered cytokine profile trends to that of the scratches that closed in 36 h, albeit at lower concentrations. The reason these scratches closed faster could be due to the variations in the initial width of the scratches, where the narrower scratches would close faster. The fact that the same 11 cytokines were highlighted in all three cytokine profiles is an indication of their importance in wound closure and re-epithelialization.

Here, we identified 11 cytokines whose concentrations were significantly increased during P0SVF-enhanced wound closure by keratinocytes. These cytokines formed a well-connected signalling network, indicating that the synergy among them might impact wound closure. This notion is a departure from the currently used approaches, where the impact of secreted cytokines and growth factors is typically examined as single agents. Our data provide a rationale for considering multifactorial experimental designs to thoroughly examine the impact of these cytokines in combination verses each as single agents. The identification of cytokines that work synergistically to enhance wound healing then can be used in the clinic through the use of hydrogels and wound dressing that are impregnated with these cytokines.

We found that there is a unique crosstalk between SVF and HEKa cells, possibly through autocrine and paracrine mechanisms, that results in a new secreted cytokine profile during P0SVF-enhanced wound closure. Among these cytokines, TGF-α and CXCL5 are particularly interesting. TGF-α and its receptor have been detected in human keratinocytes before [20], while CXCL5 has been identified as a cytokine whose expression is increased during ultraviolet-induced skin damage [21]. Our data suggest that one of the consequences of the crosstalk between P0SVF cells, and keratinocytes is the increased secretion of these cytokines that could in turn enhance keratinocyte and fibroblast proliferation and migration and promote wound healing. It would be very interesting to ascertain, which cell type (SVF and/or HEKa cells) is responsible for the altered cytokine profile described here. Although some of the cytokines we have observed in this study have been previously described in the context of the immunobiology of wound healing [22], their additive or synergistic impact on wound closure has not been considered.

## Conclusion

The data presented here serves as a proof of concept that the identification of secreted factors, with a significant impact on wound closure, requires the examination of the secretome of both SVF and keratinocytes together during wound closure. Such an approach could have a higher potential for identifying clinically relevant secreted factors that accelerate wound closure, which could be used along with wound dressings, to deliver the beneficial effects of SVF cells without the requirement to obtain the cells from the patients.

### Supplementary Information

**Additional file 1**. Detailed materials and methods.

**Additional file 2**. Characterization of P0SVF cells.

**Additional file 3**. Table showing the multiplex cytokine ELISA array data

## Data Availability

All data used in this manuscript can be found in either the figures reported, or the supplementary materials.

## Abbreviations

SVF: Stromal vascular fraction
MSC: Mesenchymal stem cell
BMSC: Bone marrow-derived mesenchymal stem cell
ADSC: Adipose-derived mesenchymal stem cell
HEKa: Human epidermal keratinocyte-adult
PBS: Phosphate-buffered saline
P0SVF-CM: Passage 0 stromal vascular fraction-conditioned medium
CM: Conditioned media
TGFα: Transforming growth factor alpha
IL6, IL8: Interleukin-6, Interleukin-8
G-CSF: Granulocyte colony-stimulating factor
