# Correction: Paracrine signalling between keratinocytes and SVF cells results in a new secreted cytokine profile during wound closure

**DOI:** 10.1186/s13287-023-03566-3

**Published:** 2023-11-10

**Authors:** Stefan Balko, Evan Kerr, Ed Buchel, Sarvesh Logsetty, Afshin Raouf

**Affiliations:** 1 Department of Immunology, Rady Faculty of Health Sciences, University of Manitoba, Winnipeg, MB Canada; 2 CancerCare Manitoba Research Institute, CancerCare Manitoba, Winnipeg, MB Canada; 3 Max Rady College of Medicine, Rady Faculty of Health Sciences, University of Manitoba, Winnipeg, MB Canada; 4 Department of Surgery, Rady Faculty of Health Sciences, University of Manitoba, Winnipeg, MB Canada

**Correction: Stem Cell Research & Therapy (2023) 14:258** 10.1186/s13287-023-03488-0

The original article [1] mistakenly misspelled Sarvesh Logsetty’s surname as ‘Logsetti’. This has since been amended.
