# Supplementary material for: Paracrine signalling between keratinocytes and SVF cells results in a new secreted cytokine profile during wound closure
Source: Stem Cell Res Ther. 2023 Sep 19;14:258. doi: 10.1186/s13287-023-03488-0 (PMC10510163; doi:10.1186/s13287-023-03488-0)
Supplement: Supplementary file 1 — Additional file 1. Detailed materials and methods. [file 13287_2023_3488_MOESM1_ESM.docx]

**Materials and Methods**

*SVF Isolation and Culture*

Excess adipose tissue samples from abdominal fat flaps were collected from patients undergoing breast reconstructive surgery, which were then enzymatically and mechanically dissociated, to yield SVF cells as previously described[1]. The fat grafts were collected based on written patient consent and by the approval of the University of Manitoba research Ethics Board (REB# HS24840). Briefly, adipose tissue was minced and dissociated overnight with collagenase and hyaluronidase. The released cells from the fat graft were immunomagnetically depleted of hematopoietic and endothelial cell lineages using the cluster of differentiation (CD) 45 and CD31 antibodies, conjugated to biotin[1]. These lineage depleted, non-passaged (P0) stromal vascular cells were denoted as P0SVF, and used for different experiments.

*Primary epidermal keratinocytes Culture*

Human Epidermal Keratinocyte – adult (HEKa) cells, harvested from human foreskin, were obtained from ThermoFisher (CAT#C005C). HEKa cells were placed in 10cm tissue culture plates with keratinocyte growth medium 2 (PromoCell, Cat# C-20111) and kept in a humidified incubator at 37°C with 5% CO_2_ with media changes every second day. Upon 80% confluence, cells were passaged with Acutase™ and were used up to passage 5.

*Colony forming Unit-fibroblast assay*

The colony forming unit-fibroblast (CFU-F) frequency in the P0SVF cells was determined as previously described[1]. Briefly, P0SVF cells were made into a single-cell suspension and placed in 6-well tissue culture plates at 1000 cells/well, and placed in a humidified incubator at 37°C and 5% CO2. After 7-10 days the cells were fixed using a mixture of Acetone and Methanol (1:1 vol/vol). The fibroblast colonies were then stained with a 0.5% Crystal Violet solution, and the number of colonies, consisting of 50 or more cells, were counted using an inverted microscope and used to calculate the CFU-F frequency of each P0SVF[2–6] (Sup. Figure 1A,B).

*Tri-Lineage Differentiation potential of ADSCs*

P0SVF cells were isolated from 3 fat graft samples, made into single-cells, and plated into 24 well plates to assess their tri-lineage differentiation potentials, as previously described[1]. Once confluent, growth media was replaced with either adipogenic (MesenCult Adipogenic Differentiation Kit CAT# 05412), chondrogenic (StemPro Chondrogenesis Differentiation Kit CAT# A1007101) or osteogenic (StemPro Osteogenesis Differentiation Kit CAT# A1007201) differentiation medium. Cells were grown as per manufacturer’s instructions and fixed with 4% Formaldehyde solution after 14 days (chondrogenic and adipogenic) or 21 days (osteogenic) to allow for the differentiation of ADSCs. The fixed cells were stained with Oil Red O, Alician Blue and Alizarian Red to detect mature adipocytes, chondrocytes and osteocytes respectively. The extent of ADSC differentiation into each lineage was documented with digital images obtained using an inverted tissue culture microscope (Sup. Figure 1C).

*Transwell Inserts*

P0SVF cells were turned into single-cell suspensions and 20,000 cells were placed in each transwell insert (Cell Quart Cat#9320412), on a membrane impermeable to cells. Tanswell inserts were then placed into each well of a 24-well tissue culture plate, containing 600µL of MSC growth medium (MesenCult Proliferation Kit, CAT# 05411). 100 µL of MSC growth medium was added to the upper chamber of the transwell inserts and cells were incubated and allowed to adhere and proliferate over the membrane surface (3-5 days). After 3-5 days, the inserts were taken out of the 24-well plate, washed with Phosphate Buffered Saline (PBS) and were then used for either obtaining P0SVF conditioned medium, or were placed in a scratch assay with HEKa cells.

*Scratch assay*

HEKa cells were placed in 24-well plates (60,000/well) with keratinocyte growth medium and allowed to grow to 90% confluence (3-5 days), after which their growth medium was replaced with keratinocyte basal medium (PromoCell CAT# 20211) to synchronize their cell cycle (18-24 hrs). Afterwards, a scratch was introduced in the middle of the HEKa cell monolayer with a 1000µL pipette tip and the plates were washed with PBS. At this point, the initial width of the scratches (0 hr) was documented through digital images obtained via an inverted tissue culture microscope. Subsequently, the cells were grown in either HEKa scratch medium, P0SVF conditioned medium (P0SVF-CM), or in HEKa scratch medium containing a transwell insert with P0SVF cells. The HEKa scratch medium (200µg insulin, 13.2 µg hydrocortisone, 400 µg transferrin, 0.05 µg EGF and 352.8 µg CaCl_2_ per 40mL of keratinocyte basal medium (PromoCell CAT# 20211)) was devised to contain the minimum essential growth factors needed to support the survival and growth of HEKa and the SVF cells in the scratch assays. This step was necessary since the growth media from the scratch assays would be analyzed for the expression of different cytokines and growth factors. The P0SVF-CM was obtained from the transwell inserts containing SVF cells, where the MSC complete medium was removed, and the wells were supplemented with HEKa scratch medium for 72 hours.

The rate of scratch closure (i.e., wound closure) was documented by obtaining digital images every 12 hours up to 36 hours. For each time point five images were obtained. All experiments were completed in triplicate, and when 80% of scratch closure was observed, growth media (i.e., conditioned media) was collected from 3 separate SVF samples and preserved at -80c for secreted cytokine profile analysis. Each SVF sample used for cytokine analysis, was also used for wound closure analysis. For two of these P0SVF/HEKa scratch assays, 80% wound closure was observed at 36 hours while for one of the P0SVF samples, 80% wound closure was observed after 24 hours.

*ELISA Assay*

Conditioned media (CM) samples from scratch assay experiments were collected and sent to Eve Technologies (Calgary, Canada, eavetechnology.com) where their Human Cytokine/Chemokine 71-Plex Discovery Assay® Array was carried out. This assay uses a high throughput Enzyme Linked Immunosorbent assay (ELISA) to detect and quantify the presence of 71 different cytokines and chemokines in the CM samples. First, cytokines with expression outside the range of their specific standard curves were omitted from the list (Sup. Table 1) after which the cytokine expressions were then averaged. Cytokine expression was then compared between the CM from scratches containing transwells, to CM from HEKa-alone or P0SVF -alone cultures. Cytokines whose level were found to be significantly increased in the P0SVF/HEKa transwells during wound closure, while also showing little or no expression in P0SVF-alone CM, were chosen for further analysis.

*Image Analysis*

Image analysis was conducted using a custom ImageJ wound healing macro, which outlines the walls of the scratch and subtracts away any cells that have migrated into the wound area, to determine the total scratch area. Wound closure at each time point was quantified by comparing the area of the scratches at every given time point, to the 0-hour images, and the data was represented as a percentage of the wound remaining open. Biological replicates were combined and the average wound closure over the 36h period was calculated.

*Statistical Analysis*

For statistical analysis of the wound closure data, an ordinary one-way ANOVA was used, with multiple comparisons to the HEKa cells alone as controls. For the cytokines of interest, one-tail, ratio paired T-Tests were conducted. Significance interval (α) was set to 95%.

**References**

1. Chatterjee S, Laliberte M, Blelloch S, Ratanshi I, Safneck J, Buchel E, et al. Adipose-Derived Stromal Vascular Fraction Differentially Expands Breast Progenitors in Tissue Adjacent to Tumors Compared to Healthy Breast Tissue. Plast Reconstr Surg. 2015;136:414e-425e.

2. Friedenstein AJ, Chailakhjan RK, Lalykina KS. THE DEVELOPMENT OF FIBROBLAST COLONIES IN MONOLAYER CULTURES OF GUINEA-PIG BONE MARROW AND SPLEEN CELLS. Cell Prolif. 1970;3:393–403.

3. Friedenstein AJ, Chailakhyan RK, Latsinik N V., Panasyuk AF, Keiliss-Borok I V. STROMAL CELLS RESPONSIBLE FOR TRANSFERRING THE MICROENVIRONMENT OF THE HEMOPOIETIC TISSUES. Transplantation. 1974;17:331–40.

4. Pochampally R. Colony forming unit assays for MSCs. Methods Mol Biol. 2008;449:83–91.

5. Delorme B, Charbord P. Culture and characterization of human bone marrow mesenchymal stem cells. Methods Mol Med. 2007;140:67–81.

6. Yang HJ, Kim K-J, Kim MK, Lee SJ, Ryu YH, Seo BF, et al. The Stem Cell Potential and Multipotency of Human Adipose Tissue-Derived Stem Cells Vary by Cell Donor and Are Different from Those of Other Types of Stem Cells. Cells Tissues Organs. 2014;199:373–83.
