# Supplementary material for: Paracrine signalling between keratinocytes and SVF cells results in a new secreted cytokine profile during wound closure
Source: Stem Cell Res Ther. 2023 Sep 19;14:258. doi: 10.1186/s13287-023-03488-0 (PMC10510163; doi:10.1186/s13287-023-03488-0)
Supplement: Supplementary file 2 — Additional file 2. Characterization of P0SVF cells. [file 13287_2023_3488_MOESM2_ESM.pdf]

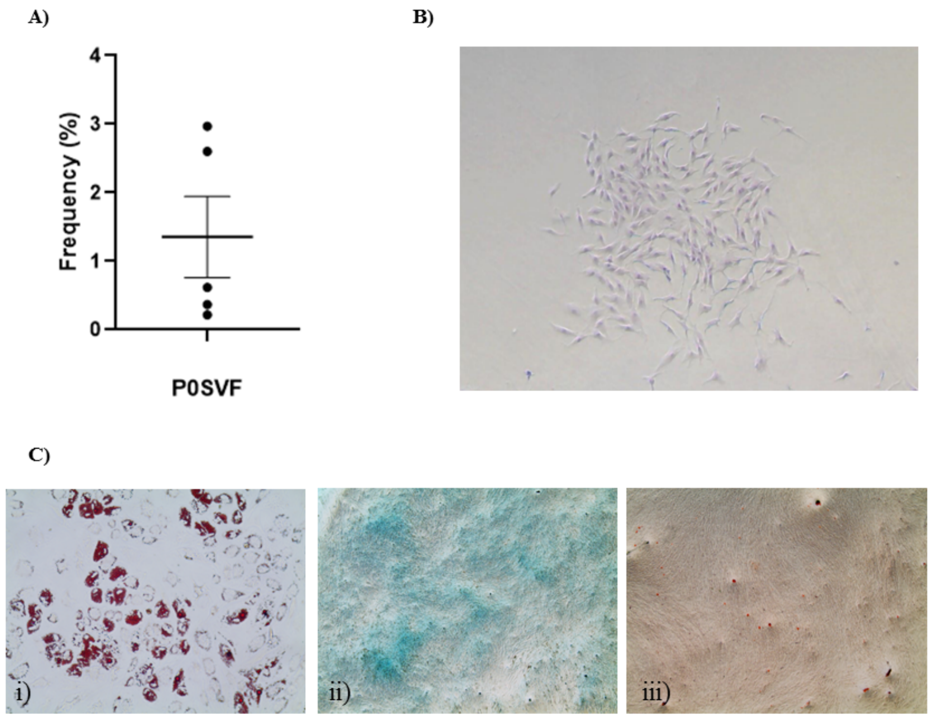

Sup. Fig 1. Characterization of P0SVF cells. A) CFU-F frequency of P0SVF cells obtained from 5 different fat graft samples were obtained and are represented as Mean ± SEM. B) Representative image of CFU-F colony. C) Representative images of trilineage differentiation of P0SVF cells into i) adipogenic, ii) chondrogenic and iii) osteogenic lineages.
