# Supplementary material for: Paracrine signalling between keratinocytes and SVF cells results in a new secreted cytokine profile during wound closure
Source: Stem Cell Res Ther. 2023 Sep 19;14:258. doi: 10.1186/s13287-023-03488-0 (PMC10510163; doi:10.1186/s13287-023-03488-0)
Supplement: Supplementary file 3 — Additional file 3. Table showing the multiplex cytokine ELISA array data [file 13287_2023_3488_MOESM3_ESM.pdf]

Sup. Table 1 Observable concentration of 71 cytokines tested on conditioned media collected from HEKa cells alone, HEKa cells with SVF in transwell inserts and SVF cells alone at 24 – 36 hr after initiation of scratches. Cytokine concentrations are represented as pg/mL.

	36h		36h			24h		
	P0SVF Sample 1		P0SVF Sample 2			P0SVF Sample 3		
	HEKa alone	HEKa w SVF	HEKa alone	HEKa w SVF	SVF alone	HEKa alone	HEKa w SVF	SVF alone
sCD40L	5.50	9.71	3.07	15.26	0.00	8.71	7.68	7.68
EGF	6.95	7.83	23.05	27.31	701.16	10.23	15.59	334.02
Eotaxin	12.75	13.44	14.06	14.96	1.90	11.40	13.69	0.00
FGF-2	21.81	22.53	27.21	30.24	11.25	24.61	28.45	0.00
FLT-3L	1.17	0.76	0.73	0.87	0.14	0.91	0.83	0.14
Fractalkine	129.17	115.66	134.67	158.87	0.00	85.60	118.20	6.48
G-CSF	9.69	282.94	9.69	240.37	0.00	6.56	13.72	0.00
GM-CSF	0.00	0.00	0.00	0.00	0.00	0.00	0.00	0.00
GROα	373.92	540.30	601.85	730.57	0.00	131.97	175.52	0.00
IFN-α2	20.71	15.81	18.99	24.87	0.00	5.95	12.48	0.00
IFNγ	2.17	1.96	1.26	1.85	0.00	1.21	1.37	0.00
IL-1α	363.63	313.03	372.20	431.53	0.00	411.75	478.84	0.00
IL-1β	1.60	2.18	2.88	3.80	0.44	1.25	1.72	0.09
IL-1RA	610.54	585.56	992.41	1492.16	0.00	507.18	678.07	0.00
IL-2	0.13	0.11	0.15	0.24	0.00	0.00	0.00	0.00
IL-3	0.42	0.24	0.21	0.22	0.00	0.01	0.11	0.46
IL-4	1.76	1.24	1.47	1.50	0.00	1.36	1.62	0.00
IL-5	0.02	0.04	0.02	0.03	0.00	0.02	0.01	0.00
IL-6	0.84	296.89	0.80	117.71	0.62	0.26	7.08	0.31
IL-7	0.00	0.00	0.00	0.00	0.00	0.00	0.00	0.00
IL-8	175.25	974.03	150.44	763.23	2.19	73.11	151.62	0.51
IL-9	4.44	3.50	3.75	4.44	0.00	1.27	3.50	0.00

IL-10	0.00	0.09	0.19	0.26	0.00	0.06	0.14	0.00
IL-12p40	2.17	3.90	4.74	5.57	2.17	5.16	4.74	0.00
IL-12p70	0.00	0.45	0.45	0.79	0.00	0.23	0.00	0.00
IL-13	39.27	33.77	42.60	36.41	0.25	37.45	49.35	0.00
IL-15	0.19	0.60	0.40	0.46	0.00	0.00	0.36	0.00
IL-17A	1.00	0.50	0.38	0.88	0.00	0.38	0.00	0.00
IL-17E/IL-25	2.08	2.08	0.35	3.00	0.00	0.00	1.44	0.00
IL-17F	2.56	2.32	3.27	3.75	0.00	0.91	1.49	0.10
IL-18	3.55	3.17	4.90	6.85	0.00	2.58	3.43	0.00
IL-22	31.96	26.27	27.79	21.44	0.00	19.78	22.99	0.00
IL-27	17.37	24.09	24.09	24.09	6.26	6.26	25.05	0.75
IP-10	10.08	3.09	3.72	2.76	0.00	4.09	3.96	0.00
MCP-1	6.29	172.79	6.61	210.83	13.96	5.34	6.29	0.19
MCP-3	9.47	9.32	13.77	32.82	0.00	8.84	11.07	0.00
M-CSF	6.45	19.13	5.86	9.38	1.79	4.99	5.13	0.00
MDC	0.84	0.47	0.94	0.72	0.00	0.18	0.33	0.00
MIG/CXCL9	0.68	0.68	0.71	1.45	0.00	0.24	0.57	0.00
MIP-1α	11.06	8.28	11.24	16.80	0.00	1.33	9.12	0.00
MIP-1β	0.00	0.00	0.00	0.00	0.00	0.00	0.00	0.00
PDGF-AA	226.45	112.62	103.42	88.41	0.00	158.12	139.77	0.00
PDGF-AB/BB	0.00	0.00	0.00	0.00	0.00	0.00	0.00	0.00
RANTES	27.50	16.86	16.46	13.58	0.00	11.83	11.41	0.00
TGFα	9.36	12.09	62.43	64.01	0.00	18.58	24.44	0.00
TNFα	5.64	5.06	3.74	3.13	0.27	3.05	3.38	0.27
TNFβ	18.18	13.03	15.41	17.18	0.18	9.21	12.14	0.00
VEGF-A	499.11	297.53	319.33	269.27	0.00	284.11	351.20	0.00
6CKine	37.67	32.07	36.77	48.29	0.00	29.03	30.07	13.70
BCA-1	0.28	0.28	0.31	0.36	0.15	0.28	0.20	0.36
CTACK	12.87	5.83	5.47	2.48	0.00	3.23	3.33	0.00

ENA-78	33.18	547.36	82.92	463.85	0.00	7.72	21.33	0.00
Eotaxin-2	15.15	8.74	14.80	20.83	0.47	4.56	8.36	0.70
Eotaxin-3	0.00	0.00	0.00	0.00	0.00	0.00	0.00	0.00
I-309	0.22	0.18	0.29	0.37	0.00	0.10	0.14	0.02
IL-16	14.70	21.19	26.66	48.38	11.06	22.08	21.64	12.96
IL-20	79.83	102.53	117.21	124.39	109.92	17.83	33.27	64.39
IL-21	0.00	0.00	0.00	0.00	0.00	0.00	0.00	0.00
IL-23	77.89	26.88	12.21	19.55	4.87	19.55	26.88	26.88
IL-28A	3.44	2.73	2.73	4.44	2.44	1.44	1.87	2.73
IL-33	0.23	0.49	0.00	0.49	0.32	0.05	0.00	0.40
LIF	0.00	0.00	0.00	0.00	0.00	0.00	0.00	0.00
MCP-2	7.49	5.69	6.78	6.86	0.00	3.39	6.22	3.39
MCP-4	49.37	15.25	14.60	20.98	0.00	9.80	13.59	0.00
MIP-1δ	14.09	4.86	19.37	24.79	4.86	2.65	4.86	2.65
SCF	5.97	5.31	3.57	2.81	0.00	3.38	4.46	0.00
SDF-1α+β	100.72	88.47	119.10	125.21	103.79	91.53	100.72	106.85
TARC	0.00	0.00	0.00	0.00	0.00	0.00	0.00	0.00
TPO	10.97	10.97	7.25	12.83	10.97	7.87	7.87	13.45
TRAIL	0.13	0.00	0.03	0.00	0.00	0.00	0.00	0.00
TSLP	0.00	0.00	0.00	0.00	0.00	0.00	0.00	0.00
